# Isolation and Characterization of Novel Sulfate-Reducing Bacterium Capable of Anaerobic Degradation of *p*-Xylene

**DOI:** 10.1264/jsme2.ME11357

**Published:** 2012-03-23

**Authors:** Yuriko Higashioka, Hisaya Kojima, Manabu Fukui

**Affiliations:** 1 The Institute of Low Temperature Science, Hokkaido University, Nishi 8, Kita 19, Kita-ku, Sapporo, Hokkaido 060–0819, Japan

**Keywords:** Sulfate-reducing bacteria, *p*-xylene, anaerobic biodegradation

## Abstract

A novel strain of *p*-xylene-degrading sulfate reducer was isolated in pure culture. Strain PP31 was obtained from a *p*-xylene-degrading enrichment culture established from polluted marine sediment. Analyses of the 16S rRNA gene and two functional genes involved in sulfate respiration and anaerobic degradation of aromatic compounds revealed that the isolate was closely related to members of the genus *Desulfosarcina*. Strain PP31 was capable of growing on *p*-xylene under sulfate-reducing conditions, and the ratio of generated sulfide and consumed *p*-xylene suggested complete oxidation by the novel isolate. The strain could not grow on benzene, toluene, ethylbenzene, *m*-xylene *o*-xylene, or *n*-hexane as an electron donor. Strain PP31 is the first isolated bacterium that degrades *p*-xylene anaerobically, and will be useful to understanding the mechanism of anaerobic degradation of *p*-xylene.

Due to its relatively high toxicity and water solubility, *p*-xylene is considered a noteworthy organic pollutant. It is a monoaromatic compound refractory to biological degradation, particularly under anoxic conditions. It has been demonstrated that enrichment cultures can anaerobically degrade *p*-xylene under nitrate- or sulfate-reducing conditions (12, 14). These studies indicated that complete oxidation of *p*-xylene can be coupled with the reduction of nitrate to N_2_ or of sulfate to sulfide. These enrichment cultures are overwhelmingly dominated by specific bacterial phylotypes, suggesting that these bacteria are responsible for the anaerobic degradation of *p*-xylene. Despite repeated trials with these cultures, a bacterium that can anaerobically degrade of *p*-xylene has not yet been isolated in pure culture. In contrast, the other isomers of xylene have been shown to be degraded by pure cultures of sulfate-reducing bacteria (4, 11).

The above-mentioned *p*-xylene-degrading sulfate-reducing culture was dominated by a specific organism related to the genus *Desulfosarcina*, which includes strains capable of degrading toluene and/or *o*-xylene. Impurity in this enrichment culture was shown in another study, by shifts in the community structure induced when the substrate was changed, and by the isolation of another strain from it (5). In the present study, the dominant organism in the enrichment culture was successfully isolated in pure culture by applying a modified isolation technique. The obtained strain was characterized in detail; this novel sulfate reducer could anaerobically degrade *p*-xylene without the assistance of other bacteria.

## Materials and Methods

### Maintenance of *p*-xylene-degrading enrichment culture

A *p*-xylene-degrading sulfate-reducing enrichment culture was previously established using marine sediments from Shuaiba, Kuwait as the inoculum (13). The enrichment culture was maintained in our laboratory by periodic transfer to fresh medium. The basal medium used was identical to that used in the previous study. It contained the following constituents (L^−1^): 20.0 g NaCl; 4 g Na_2_SO_4_; 0.2 g KH_2_PO_4_; 0.25 g NH_4_Cl; 3.0 g MgCl_2_·6H_2_O; 0.5 g KCl; 0.15 g CaCl_2_·2H_2_O; 1 mL trace element solution; 1 mL selenite-tungstate solution; 1 mL vitamin mixture solution; 1 mL vitamin B_12_ solution; 30 mL of 1 M NaHCO_3_ solution; and 1.5 mL of 1 M Na_2_S solution (18). This bicarbonate-buffered defined medium was also used for all culture experiments performed in the present study unless specified otherwise. As the sole electron donor to sustain growth, 2% (v/v) *p*-xylene solution prepared in 2,2,4,4,6,8,8-heptamethylnonane to reduce the toxic effects was added to the basal medium (14). The volumetric ratio of diluted *p*-xylene solution to basal medium was 1:25. An aliquot (1% of the volume of fresh medium) of grown enrichment culture was inoculated using a N_2_-flushed sterilized syringe into freshly prepared medium. As an additional reductant, sodium dithionite was added to the fresh medium prior to each inoculation. The culture bottles were incubated at 28°C in the dark. They were manually shaken several times a week. Growth was monitored by turbidity and dissolved sulfide concentration in the aqueous phase, determined as described previously (1).

### Isolation of a novel sulfate-reducing bacterium

**Fig. 1:**
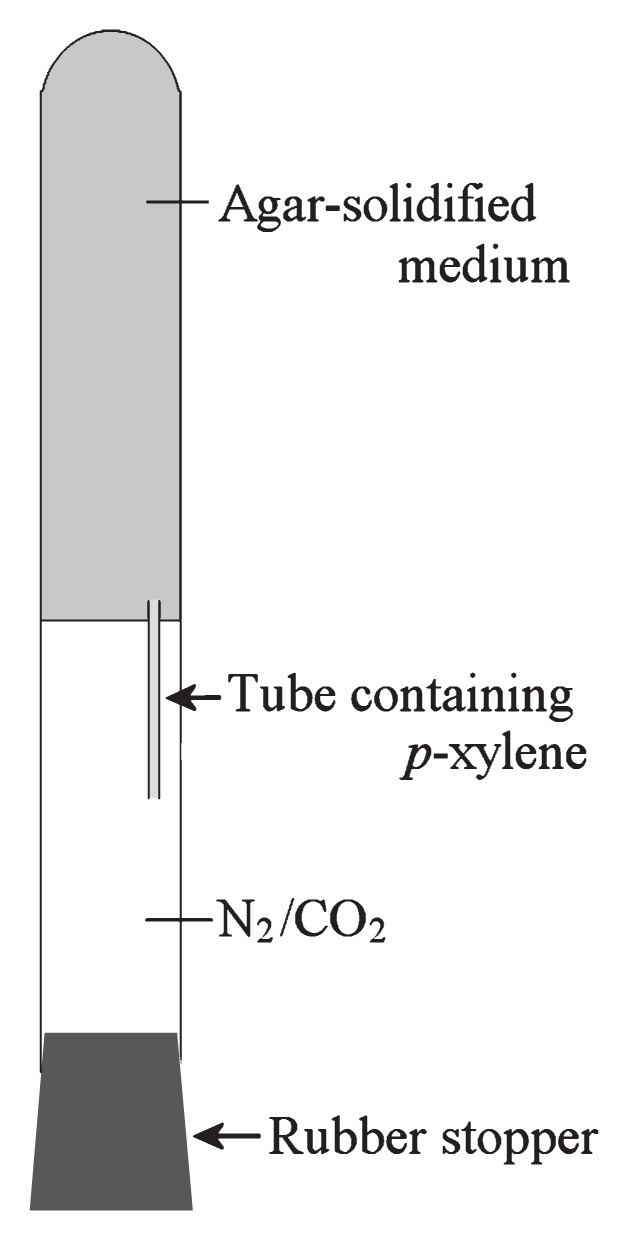
Schematic illustration of method used to isolate *p*-xylene-degrading sulfate reducer.

The *p*-xylene-degrading enrichment culture was subjected to agar shake dilution (18) with some modification, with *p*-xylene as the sole substrate for growth. First, the usual agar shake dilution was performed using basal medium containing no water-soluble organic substrate. After solidification of the medium, a small glass tube (internal diameter, 1 mm; length, 40 mm) was stuck to the surface of solidified medium in each agar shake tube (Fig. 1). Each small tube contained a droplet of 2%-diluted (v/v) *p*-xylene solution, which was held in the tube by capillarity. The headspace of the agar shake tubes was filled with the N_2_/CO_2_ (80:20 [v/v]) mixture, and the tubes were incubated at 28°C. After 5 months, an isolated colony was picked and inoculated into fresh medium containing 5 mmol L^−1^ acetate. The purity of the resulting culture was confirmed by the experiments described below. The isolate was designated as strain PP31.

### Physiological characterization of the isolated strain PP31

The physiological characterization of strain PP31 was performed at 28°C. The utilization of electron donors in the presence of sulfate was tested by monitoring the growth in media, each containing one of the following substrates (concentrations in mmol L^−1^ unless otherwise stated): propionate (5), succinate (5), formate (5), fumarate (10), pyruvate (10), *n*-butyrate (5), lactate (20), acetate (10), benzoate (1), glucose (10), ethanol (5), phenol (0.5), and yeast extract (0.5 g L^−1^). For the test hydrocarbon utilization, toluene, benzene, *o*-xylene, *m*-xylene, *p*-xylene, ethylbenzene, and *n*-hexane were diluted to 2% (v/v) in 2,2,4,4,6,8,8-heptamethylnonane. Each solution was added to 25-fold volume of basal medium. In the H_2_ utilization test, the headspace of the culture bottle was filled with H_2_:N_2_:CO_2_ (50:40:10 by volume, total pressure=200 kPa). The utilization of electron acceptors was tested using sulfate-free basal medium containing 5 mmol L^−1^ propionate as an electron donor. The electron acceptors tested were thiosulfate (10 mmol L^−1^), nitrate (10), and sulfite (5). Growth under fermentative conditions was also tested with sulfate-free basal medium, using fumarate or lactate as substrates. The optimum growth temperature was determined by incubation at 8 different temperatures ranging from 13 to 42°C, using the basal medium supplemented with 10 mmol L^−1^ fumarate.

All culture experiments performed to allow physiological characterization were followed by denaturing gradient gel electrophoresis (DGGE) analysis to verify the identity and purity of the cells grown under various conditions. Cells were harvested from growth-positive cultures using centrifugation and washed with phosphate-buffered saline. From the collected cells, partial fragments of the 16S rRNA gene were directly amplified using the primer pair GC341F/533r (10, 12). DGGE was performed as described previously (5).

### Anaerobic degradation of *p*-xylene by PP31

Portions (1.2 mL) of the pure culture of strain PP31 grown on 2% *p*-xylene were inoculated into bottles containing 115 mL of the fresh medium and 5 mL *p*-xylene solution of various concentrations. To investigate the effect of *p*-xylene concentration on the strain, solutions of various concentrations (0, 0.2, 0.5, 1, 2, 4, 6, 8, 10, and 15% in v/v) were prepared by diluting *p*-xylene with 2,2,4,4,6,8,8-heptamethylnonane. During incubation at 28°C, the sulfide concentration of each bottle was continually monitored as a readily measurable indicator of *p*-xylene degradation. The concentration of *p*-xylene in the carrier phase was investigated by a gas chromatography system (GC-2014; Shimadzu, Kyoto, Japan) equipped with a flame ionization detector. The hydrophobic phase directly taken from the cultures was diluted with acetone, and then mixed with a known amount of ethylbenzene as an internal standard. Hydrocarbons in the diluted solution were separated by DB-5MS column (J & W Scientific, Flosom, CA, USA; length, 30 m; internal diameter, 0.25 mm). The oven temperature program for analysis was begun at 50°C for 10 min, proceeded at the rate of 5°C min^−1^ to 250°C, and held for 60 min. The injector and detector temperatures were both set at 250°C.

### Phylogenetic analysis and G+C content determination

The total DNA of strain PP31 was extracted using the method described previously (19). From the extracted genomic DNA, fragments of nearly the full length of the 16S rRNA gene were amplified with primers 27f and 1492r (10). Amplification was initiated with 1 min of denaturation at 94°C. Each thermal cycle consisted of the following steps; 30 s denaturation at 94°C, 30 s annealing at 55°C, and 60 s elongation at 72°C. Twenty-three cycles were carried out, followed by an additional extension of 10 min at 72°C. To confirm the purity of the culture, partial fragments of the 16S rRNA gene were also amplified using three universal PCR primer pairs, 27f/533r, 515f/1492r, and 341f/907r (10, 12).

Phylogenetic analyses were also performed on two functional genes, *dsrA* and *bamA*, which are involved in sulfate respiration and anaerobic degradation of aromatic compounds, respectively. Fragments of the *dsrA* gene that encodes the alpha subunit of dissimilatory sulfite reductase were amplified using primers DSR1Fdeg (7) and DSR1334R (16). The PCR steps were as follows: initial denaturation for 3 min at 94°C; 30 cycles of denaturation (40 s at 94°C), annealing (40 s at 54°C), and extension (120 s at 72°C); and a final extension for 8 min at 72°C. Fragments of the *bamA* gene that encoded 6-oxocyclohex-1-ene-1-carbonyl-coenzyme A hydrolase were amplified using the primer pair BamA-SP9/BamA-ASP1 (8). The PCR steps were as follows: initial denaturation for 10 min at 94°C; 35 cycles of denaturation (30 s at 94°C), annealing (45 s at 60°C), extension (1 min at 72°C), and a final extension for 10 min at 72°C. For analysis of the *bamA* gene, one of the closest relatives of PP31 was also sequenced as a reference. An actively growing culture of *Desulfosarcina variabilis* DSM 2060^T^ was obtained from Deutsche Sammlung von Mikroorganismen und Zelkulturen (DSMZ) and analyzed using a method identical to that used for strain PP31.

The PCR products were treated with ExoSAP-IT (USB, Cleveland, OH, USA) and then used as templates for direct sequencing. The sequences of the PCR products were determined by cycle sequencing using a dye terminator (BigDye Terminator v3.1 Cycle Sequencing Kit; Applied Biosystems, Foster City, CA, USA).

The nucleotide sequence of the 16S rRNA gene and amino acid sequences deduced from the nucleotide sequences of the *dsrA* and *bamA* genes were aligned with reference sequences from the public database using the ClustalX program (17). The phylogenetic tree was constructed by the neighbor-joining method using MEGA software version 3.1 (9). Bootstrap analysis was performed for 500 replicates.

Genomic DNA for G+C content determination was extracted with the AquaPure genomic DNA isolation kit (Bio-Rad, Hercules, CA, USA). The extracted DNA was digested with P1 nuclease using a commercially available kit (Yamasa GC kit; Yamasa Shoyu, Choshi, Japan) according to the manufacturer’s instructions. The resulting mixture of deoxyribonucleosides was analyzed with a high-performance liquid chromatography system equipped with an ODS column (YMC-Pack ODS-AQ; YMC, Kyoto, Japan) and a UV detector (SPD-20A; Shimadzu, Kyoto, Japan). The G+C content was determined by using an equimolar mixture of four deoxyribo-nucleosides supplied in the kit as the quantitative standard.

### Nucleotide sequence accession numbers

The nucleotide sequences obtained in this study have been assigned DDBJ/EMBL/GenBank accession numbers AB610146–AB610148 (16S rRNA, *dsrA*, and *bamA* genes of strain PP31) and AB610408 (*bamA* gene sequence of *D. variabilis*).

## Results

### Isolation of a novel sulfate-reducing bacterium, strain PP31

As a result of agar shake dilution, with *p*-xylene as the sole substrate, well-isolated colonies were obtained from the deep agar tube. A culture originating from one of these colonies was subjected to further experiments to check its purity. First, genomic DNA was extracted from cells in the culture for analysis of the 16S rRNA gene. The PCR product obtained with primer pair 27f/1492r was fully sequenced without ambiguity. It perfectly matched the sequence of the DGGE band pXy-K-13 (13), thus corresponding to the dominant organism in the original enrichment culture. All PCR products obtained using three universal primer pairs were also sequenced without ambiguity and resulting sequences were consistent with that obtained with the 27f/1492r primer pair.

The previous study on enrichment indicated that the organism corresponding to DGGE band pXy-K-13 was readily outcompeted by other bacteria in the presence of aqueous substrates (5). To confirm the absence of bacterial contaminants, the culture obtained in this study was transferred to medium containing various electron donors or acceptors including those less selective. As described below, no contaminating organism was detected in these experiments. Based on these results, it was concluded that the dominant bacterium in the enrichment culture was isolated in pure culture, and designated as strain PP31.

### Degradation of *p*-xylene by strain PP31

**Fig. 2:**
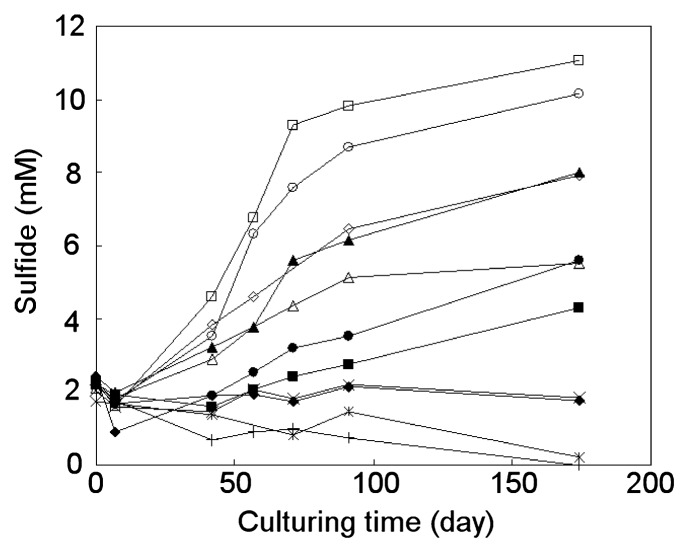
Changes in sulfide concentration in culture of PP31 incubated in the presence of various concentrations of *p*-xylene in the carrier phase. ^*^, 0%; △, 0.2%; ○, 0.5%; □, 1%; ⋄, 2%; ▲, 4%; ●, 6%; ■, 8%; ◆, 10%; +, 15%; ×, abiotic control with 2% *p*-xylene.

**Fig. 3:**
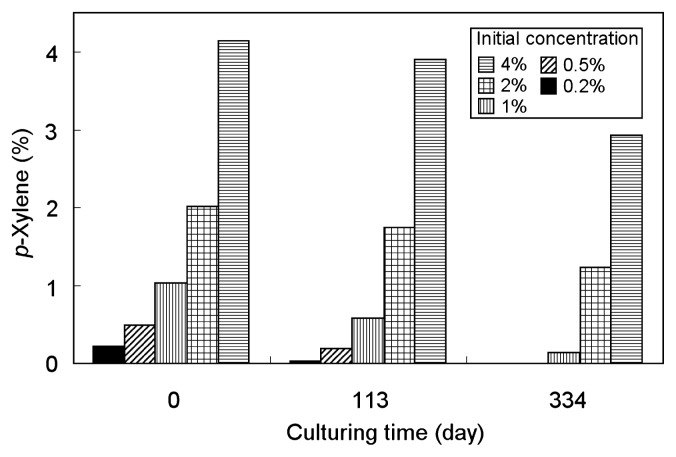
Changes in *p*-xylene concentrations in carrier phase in cultures of strain PP31. The experiment was started on day 0 with 0.2, 0.5, 1, 2, and 4% *p*-xylene.

The pure culture of strain PP31 was transferred to medium not supplemented with any water-soluble substrate but containing *p*-xylene. With 2% *p*-xylene in the carrier phase, growth of the strain was consistently observed when successively transferred to the same *p*-xylene-containing medium. The effect of *p*-xylene concentration on the degradation activity of PP31 was investigated using various concentrations of *p*-xylene. The resulting changes in the sulfide concentration are shown in Fig. 2. These cultures used *p*-xylene as the sole electron donor and sulfate as the sole electron accepter; therefore, an increase in sulfide indicated oxidative degradation of *p*-xylene coupled with sulfate reduction. Continuous sulfide production was observed in cultures at *p*-xylene concentrations of 8% or lower (Fig. 2). In these cultures, turbidity, indicating bacterial growth, was also observed. Degradation of *p*-xylene was confirmed by direct measurement of its concentration in the carrier phase. *p*-Xylene was quantified for cultures of 5 selected concentrations, and a decrease in *p*-xylene concentration was consistently observed (Fig. 3). With 0.2 and 0.5% concentrations, *p*-xylene was depleted until day 334. Total amounts of sulfide in these cultures on day 334 were compared to the amounts of consumed *p*-xylene. The calculated molar ratios of generated sulfide and consumed *p*-xylene were 5.3 and 5.0, respectively. These values are comparable to the theoretically expected value of 5.25, calculated assuming complete oxidation of *p*-xylene coupled with sulfate reduction to sulfide, as follows:

$${\text{C}}_{6}{\text{H}}_{4}{\left({\text{CH}}_{3}\right)}_{2}+5.25{{\text{SO}}_{4}}^{2-}\to 8{\text{CO}}_{2}+5.25{\text{S}}^{2-}+5{\text{H}}_{2}\text{O}.$$

### Phylogenetic and physiological characteristics of strain PP31

**Fig. 4:**
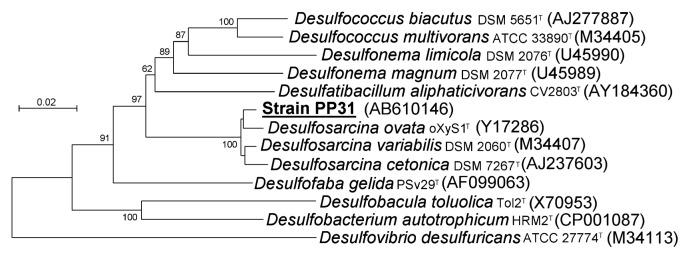
Phylogenetic position of strain PP31 within the family *Desulfobacteraceae*, based on 16S rRNA gene sequence analysis. *Desulfovibrio desulfuricans* is included as an outgroup. The tree was constructed with the neighbor-joining method using 1256 positions. Genetic distances were calculated using the Kimura 2-parameter model. Numbers on nodes represent percentage values of 1,000 bootstrap resampling (values larger than 50 are shown).

**Fig. 5:**
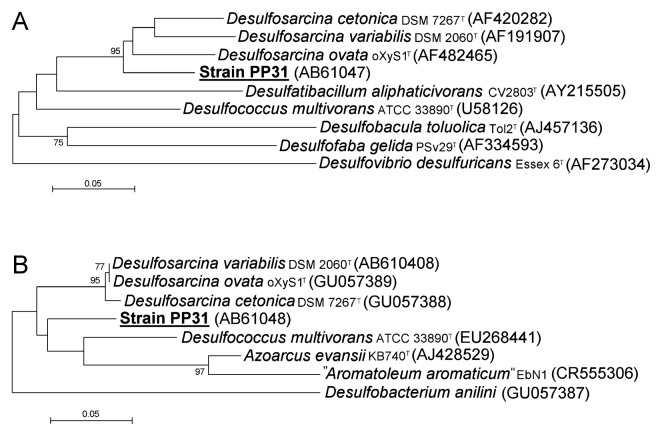
Phylogenetic position of strain PP31 based on partial sequences of *dsrA* gene (A) and *bamA* gene (B). These neighbor-joining trees were constructed with amino acid sequences deduced from nucleotide sequences (218 and 83 amino acid positions were used for DsrA and BamA, respectively). Numbers on nodes are percentage values of 1,000 bootstrap resampling (values larger than 50 are shown). Genetic distances were calculated with the Poisson correction

Phylogenetic analysis based on the nearly full-length 16S rRNA gene sequence indicated that strain PP31 belongs to the class *Deltaproteobacteria* (Fig. 4). It is most closely related to members of the genus *Desulfosarcina* shown in the tree, with sequence identity of 98%. The relatedness of PP31 to *Desulfosarcina* species was also supported by analyses of functional genes (Fig. 5). The sequence of the *bamA* gene in *D. variabilis* was not available in the public database and was therefore determined in the present study. The nucleotide sequence obtained was similar to that of *Desulfosarcina ovata*, and the amino acid sequence deduced was identical to that of *D. ovata*. These sequences were also closely related to that of *Desulfosarcina cetonica*; however, strain PP31 was distinct from these species.

Genomic G+C content of strain PP31 was determined to be 54.8%, which is higher than those of *Desulfosarcina variabilis* and *Desulfosarcina ovata* (51%), but lower than *Desulfosarcina cetonica* (59%).

In the presence of sulfate, cell growth was observed in cultures containing the following substrates: acetate, pro-pionate, succinate, pyruvate, formate, fumarate, *n*-butyrate, benzoate, and yeast extract. Autotrophic growth on hydrogen was also observed. No cell growth was observed in basal medium containing any of the following substrates: lactate, phenol, ethanol, and glucose. Similarly, no growth was observed in the cultures with hydrocarbons other than *p*-xylene. As for an electron acceptor alternative to sulfate, growth was observed with thiosulfate, but not with nitrate or sulfite. In sulfate-free medium without an alternative electron acceptor, fumarate supported the growth of strain PP31, but lactate did not. The growth of strain PP31 was observed over the temperature range of 20–37°C, with the optimum of 28°C.

As part of the purity check, growth-positive cultures in these experiments were subjected to DGGE. In all cases, only a single band at the same position as the inoculum was observed (supplemental material), indicating successful isolation of the dominant organism in the original enrichment culture.

## Discussion

Benzene, toluene, ethylbenzene, and three isomers of xylene are often collectively referred to as BTEX. Among the BTEX compounds, only *p*-xylene has not yet been demonstrated to be anaerobically degraded by the activity of a single organism. To our knowledge, strain PP31 is the first isolated bacterium that degrades *p*-xylene anaerobically.

To reveal the biodegradation mechanism of hazardous materials, it is important to obtain pure cultures of organisms that have the ability to catabolize the target materials. Although the importance of anaerobic processes has been widely recognized, difficulties in the cultivation and isolation of anaerobes have hindered advances in the study of the anaerobic biodegradation of pollutants. In recent years, exhaustive analyses of genomes and proteomes using advanced techniques have been applied to pure culture of strict anaerobes capable of degrading hydrocarbons (2, 3). In this context, the development of a pure culture of strain PP31 potentially has great importance in the field of bioremediation.

It is likely that the isolated strain PP31 belongs to the genus *Desulfosarcina*. Members of the genus *Desulfosarcina* are sulfate-reducing bacteria capable of degrading a variety of complex organic substances. In this genus, three species with valid published names are known, two of which, *D. ovata* and *D. cetonica*, are able to use toluene as an electron donor; the former can also degrade *o*-xylene. In addition, another toluene-degrading bacterium, related to these species, has been recently isolated (6). In contrast to these close relatives, strain PP31 utilized *p*-xylene as an electron donor to sustain growth but was not able to use other BTEX compounds including *o*-xylene and toluene. Comparative analysis of these bacteria with different functions may be an effective approach to understand the mechanism of anaerobic degradation of monoaromatic hydrocarbons coupled with sulfate reduction or other types of anaerobic respiration.

## Supplementary Material
