# Supplementary material for: Isolation and Characterization of Novel Sulfate-Reducing Bacterium Capable of Anaerobic Degradation of p-Xylene
Source: Microbes Environ. 2012 Mar 23;27(3):273–7. doi: 10.1264/jsme2.ME11357 (PMC4036038; doi:10.1264/jsme2.ME11357)
Supplement: Supplementary file 1 [file 27_273_s1.pdf]

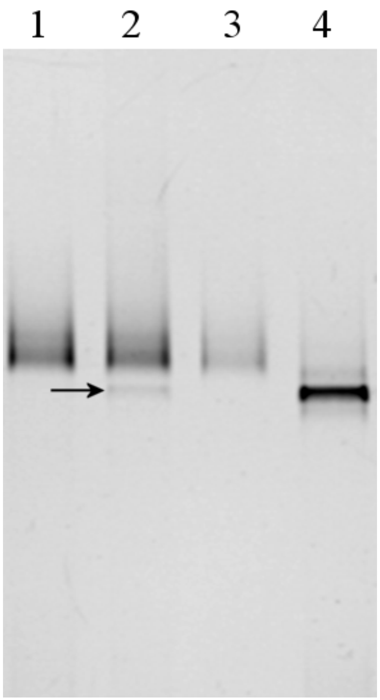

Fig. S1 DGGE profiles of 16S rRNA gene fragments indicating purity of the culture of strain PP31 obtained in the present study. For comparison, results of the original enrichment culture and another strain isolated in a previous study are also shown. Lane 1, original *p*-xylene-degrading enrichment culture; lane 2, enrichment culture transferred to the benzoate-containing medium; lane 3, culture of strain PP31 grown on benzoate; lane 4, pure culture of strain PL12. As reported previously, the original enrichment culture was dominated by a single phylotype (lane 1), but the presence of another phylotype (indicated by an arrow) become apparent in presence of aqueous substrate (lane 2). This minor member has been isolated as strain PL12 (lane 4, see Higashioka et. al, 2009 for detail). The strain PP31 corresponds to the dominant organism in the original enrichment culture (lane 3), and no additional band was observed even under the presence of a less selective substrate.
